# Supplementary material for: Associations between Greenspace and Gentrification-Related Sociodemographic and Housing Cost Changes in Major Metropolitan Areas across the United States
Source: Int J Environ Res Public Health. 2021 Mar 23;18(6):3315. doi: 10.3390/ijerph18063315 (PMC8005168; doi:10.3390/ijerph18063315)
Supplement: Supplementary file 1 [file ijerph-18-03315-s001.pdf]

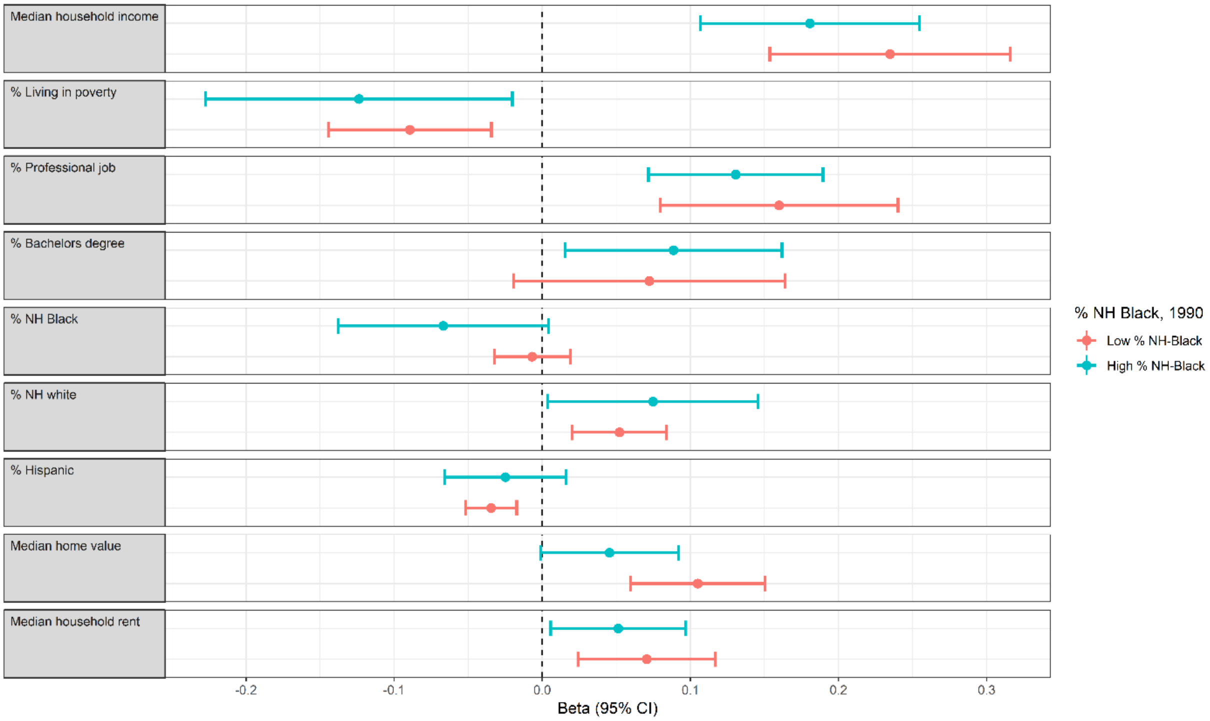

Figure S1. Meta-analytic estimates of association between % greenspace in 1992 and sociodemographic and housing cost changes, 1990-2000, among gentrifiable census tracts in the 43 largest MSAs in the United States, adjusted for population density in 1990 and stratified by % non-Hispanic Black in year 1990.

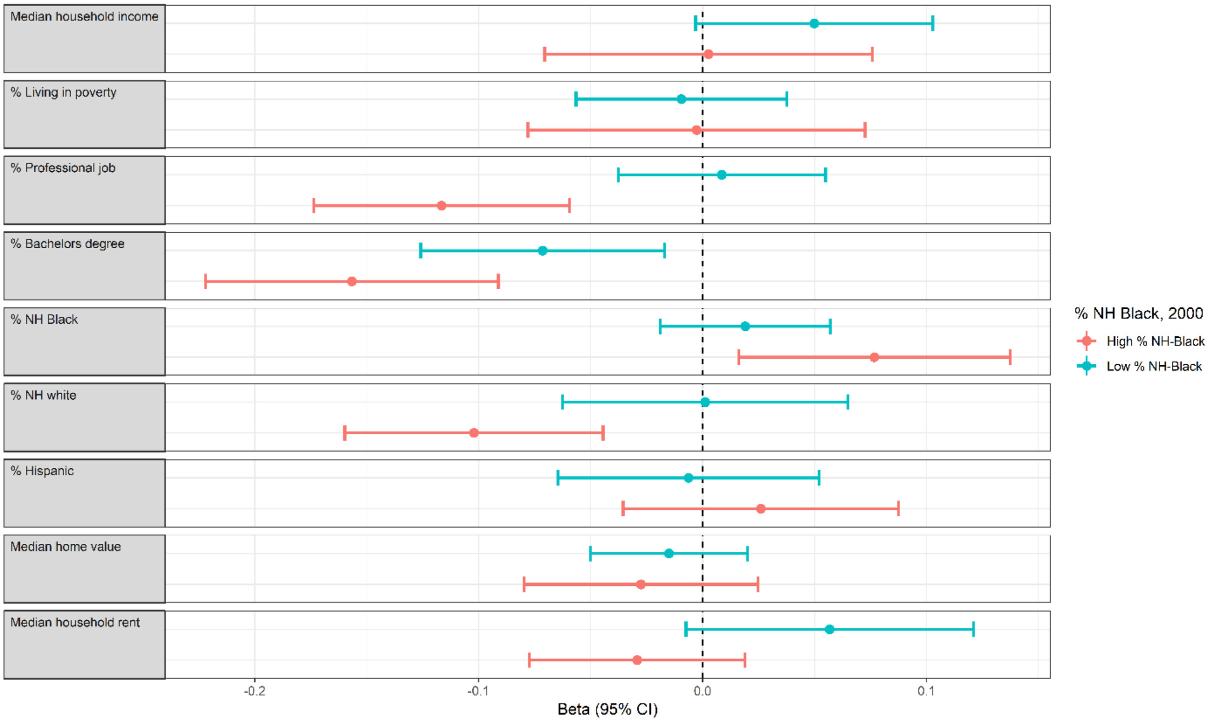

Figure S2. Meta-analytic estimates of association between % greenspace in 2001 and sociodemographic and housing cost changes, 2000-2010, among gentrifiable census tracts in the 43 largest MSAs in the United States, adjusted for population density in 2000 and stratified by % Black in year 2000.

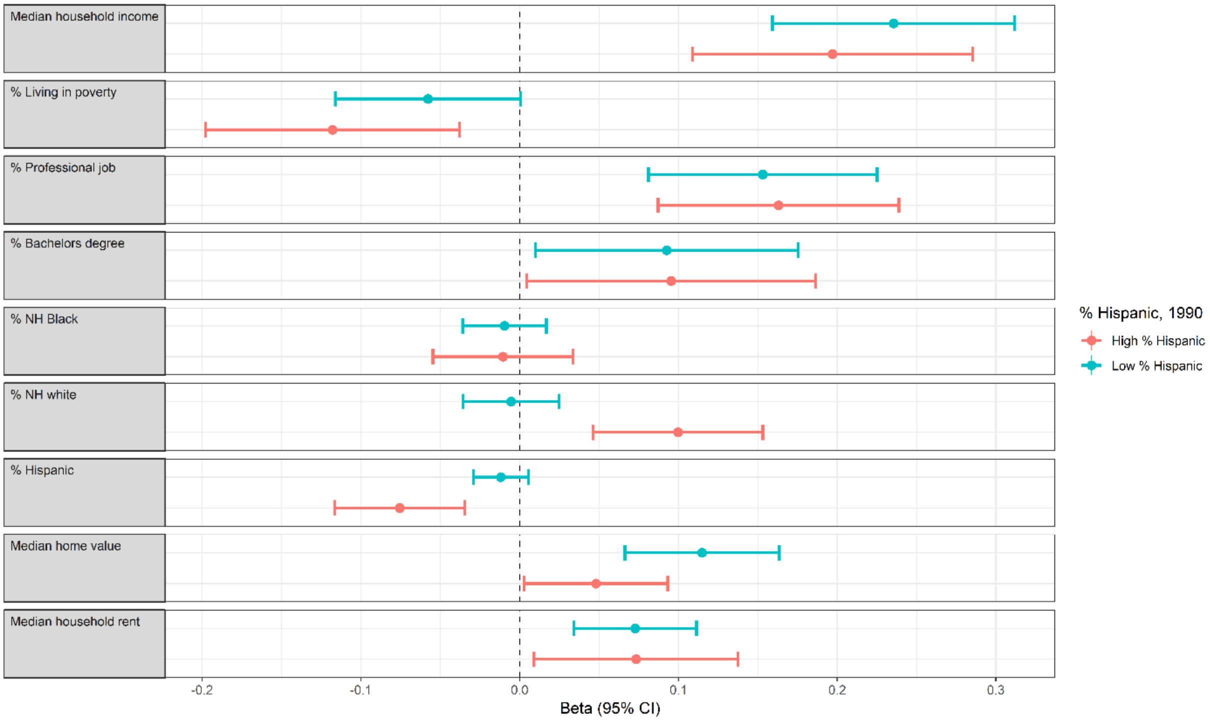

Figure S3. Meta-analytic estimates of association between % greenspace in 1992 and sociodemographic and housing cost changes, 1990-2000, among gentrifiable census tracts in the 43 largest MSAs in the United States, adjusted for population density in 1990 and stratified by % Hispanic in year 1990.

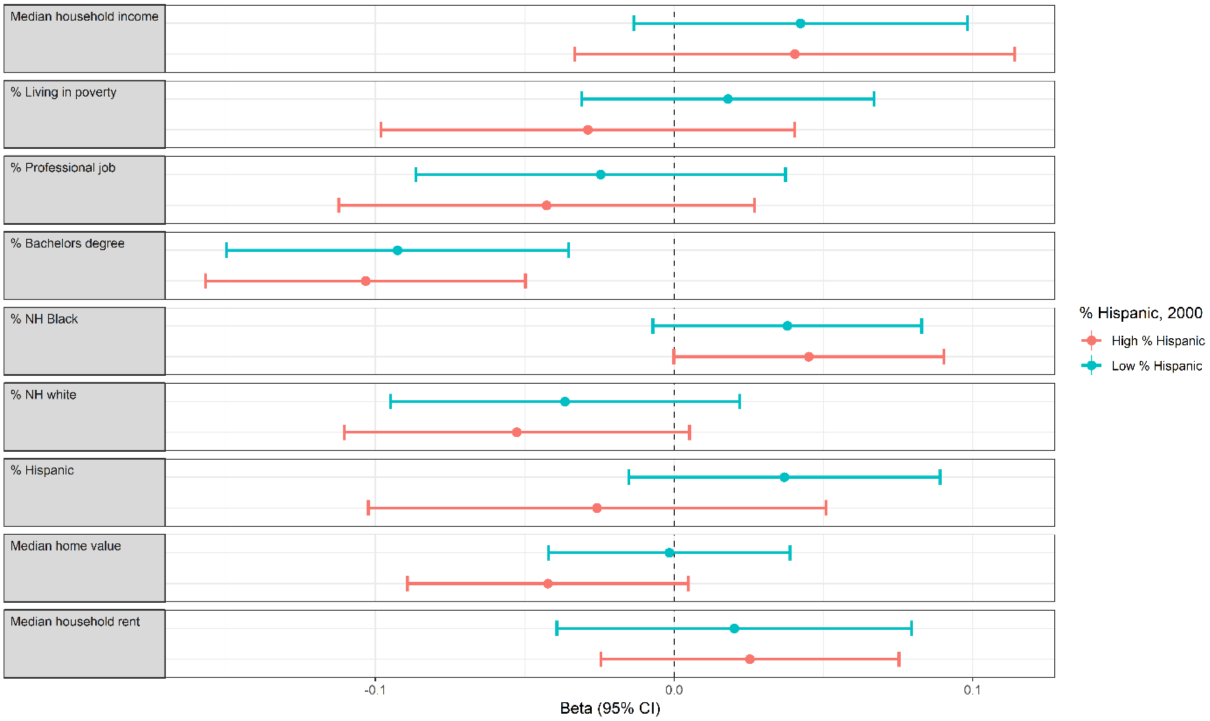

Figure S4. Meta-analytic estimates of association between % greenspace in 2001 and sociodemographic and housing cost changes, 2000-2010, among gentrifiable census tracts in the 43 largest MSAs in the United States, adjusted for population density in 2000 and stratified by % Hispanic in year 2000.

Table S1. Descriptive statistics for the gentrifiable census tracts within each metropolitan statistical area included in the 1990-2000 period analysis

				% Green 1992					Population density, 1990					% Non-Hispanic Black, 1990					% Hispanic, 1990				
MSA	MSA Name	Core city name	Number of gentrifiable census tracts	Q1	Q2	Q3	Min	Max	Q1	Q2	Q3	Min	Max	Q1	Q2	Q3	Min	Max	Q1	Q2	Q3	Min	Max
12060	Atlanta-Sandy Springs-Roswell, GA	Atlanta	708	0.41	0.62	0.93	0.03	1	121.03	501.63	1115.82	5.68	5585.74	0.05	0.14	0.44	0	1	0.01	0.01	0.02	0	0.32
12420	Austin-Round Rock, TX	Austin	258	0.13	0.56	0.93	0	1	84.37	550.91	1594.43	2.26	7392.66	0.03	0.06	0.09	0	0.87	0.11	0.17	0.29	0.04	0.88
12580	Baltimore-Columbia-Towson, MD	Baltimore	505	0.09	0.24	0.54	0	1	766.7	1873.54	4168.17	13.06	38082.5	0.03	0.11	0.51	0	0.99	0.01	0.01	0.02	0	0.1
14460	Boston-Cambridge-Newton, MA-NH	Boston	745	0.05	0.15	0.49	0	0.95	647.42	2151.01	5652.35	20.54	35281.02	0.01	0.02	0.05	0	0.92	0.01	0.02	0.07	0	0.81
16740	Charlotte-Concord-Gastonia, NC-SC	Charlotte	398	0.37	0.72	0.91	0.03	0.99	77.97	244.24	720.31	7.36	3211.14	0.06	0.14	0.26	0.01	0.99	0	0.01	0.01	0	0.13
16980	Chicago-Naperville-Elgin, IL-IN-WI	Chicago	1654	0.03	0.1	0.35	0	1	1164.29	2722.46	6161.79	6.76	190809.19	0	0.03	0.33	0	1	0.02	0.05	0.14	0	0.97
17140	Cincinnati, OH-KY-IN	Cincinnati	372	0.19	0.44	0.86	0	1	272.11	1206.66	2326.23	9.35	9442.64	0.01	0.02	0.13	0	0.99	0	0	0.01	0	0.03
17460	Cleveland-Elyria, OH	Cleveland	474	0.03	0.11	0.38	0	0.99	1017.94	2022.23	3412.19	16.8	9372.58	0.01	0.04	0.47	0	0.99	0.01	0.01	0.02	0	0.62
18140	Columbus, OH	Columbus	324	0.13	0.38	0.87	0.01	1	200.47	1224.94	2238.27	7.79	10232.01	0.01	0.04	0.13	0	0.94	0	0.01	0.01	0	0.04
19100	Dallas-Fort Worth-Arlington, TX	Dallas	975	0.06	0.29	0.72	0	1	265.24	1088.71	1891.96	1.39	13546.02	0.03	0.06	0.15	0	0.99	0.05	0.08	0.16	0	0.94
19740	Denver-Aurora-Lakewood, CO	Denver	447	0.06	0.15	0.37	0	1	784.97	1613.9	2234.08	0.35	8449.94	0.01	0.01	0.06	0	0.92	0.05	0.08	0.15	0.01	0.82
19820	Detroit-Warren-Dearborn, MI	Detroit	960	0.03	0.11	0.43	0	1	907.07	1913.52	2918.32	14.7	6341.23	0	0.02	0.6	0	0.99	0.01	0.01	0.02	0	0.54
26420	Houston-The Woodlands-Sugar Land, TX	Houston	796	0.13	0.33	0.65	0	0.99	413.09	1213.75	2060.96	3.15	12664.1	0.03	0.1	0.27	0	0.99	0.09	0.17	0.3	0	0.96
26900	Indianapolis-Carmel-Anderson, IN	Indianapolis	297	0.12	0.32	0.74	0	1	407.01	931.62	1622.79	9.09	5004.75	0	0.03	0.2	0	0.99	0.01	0.01	0.01	0	0.03
28140	Kansas City, MO-KS	Kansas City	386	0.12	0.34	0.81	0	1	198.7	961.63	1588.39	4.21	5059.37	0.01	0.03	0.2	0	0.98	0.01	0.02	0.03	0	0.67
29820	Las Vegas-Henderson-Paradise, NV	Las Vegas	296	0.13	0.22	0.48	0.03	1	265.36	1347.8	2261.14	0.23	8110.8	0.03	0.04	0.08	0	0.95	0.06	0.08	0.11	0.01	0.49
31080	Los Angeles-Long Beach-Anaheim, CA	Los Angeles	2178	0.06	0.11	0.18	0	1	2791.44	4032.49	6380.7	1.57	40804.46	0.01	0.03	0.09	0	0.93	0.19	0.36	0.62	0.01	0.99
31140	Louisville/Jefferson County, KY-IN	Louisville	222	0.16	0.36	0.83	0.02	0.99	381.24	1128.97	1908.52	9.91	4488.77	0.01	0.03	0.11	0	0.98	0	0.01	0.01	0	0.02
32820	Memphis, TN-MS-AR	Memphis	233	0.1	0.27	0.9	0.03	0.99	100.45	1025.08	1828.16	3.11	4445.91	0.14	0.38	0.78	0	1	0	0.01	0.01	0	0.07
33100	Miami-Fort Lauderdale-West Palm Beach, FL	Miami	894	0.03	0.08	0.21	0	0.98	1228.78	2098.43	3179.05	1.78	27548.79	0.01	0.04	0.18	0	0.99	0.06	0.13	0.43	0	0.96
33340	Milwaukee-Waukesha-West Allis, WI	Milwaukee	320	0.03	0.09	0.26	0	0.99	1180.3	2552.7	4464.5	24.23	11536.92	0	0.01	0.25	0	0.98	0.01	0.02	0.04	0	0.67
33460	Minneapolis-St. Paul-Bloomington, MN-WI	Minneapolis	584	0.08	0.21	0.68	0	1	489.36	1279.45	2241.57	3.32	8040.29	0	0.01	0.03	0	0.8	0.01	0.01	0.02	0	0.36
34980	Nashville-Davidson-Murfreesboro-Franklin, TN	Nashville	282	0.42	0.74	0.97	0.04	1	42.18	306.35	905.74	4.06	4760.29	0.03	0.07	0.14	0	0.99	0	0.01	0.01	0	0.02
35380	New Orleans-Metairie, LA	New Orleans	293	0.05	0.12	0.28	0	0.97	1041.19	2566.43	4016.91	1.92	18975.6	0.12	0.42	0.81	0	1	0.01	0.03	0.05	0	0.3
35620	New York-Newark-Jersey City, NY-NJ-PA	New York	3458	0.01	0.03	0.13	0	0.98	2680.49	8000.83	16109.9	6.3	80647.62	0.01	0.06	0.35	0	0.98	0.05	0.1	0.27	0	0.96
36420	Oklahoma City, OK	Oklahoma City	271	0.01	0.2	0.72	0	0.99	266.54	1136.6	1622.26	2.87	3079.97	0.02	0.05	0.15	0	0.98	0.02	0.03	0.05	0	0.52
36740	Orlando-Kissimmee-Sanford, FL	Orlando	291	0.14	0.3	0.56	0.03	0.98	232.45	702.59	1266.01	1.41	3955.55	0.02	0.04	0.12	0	0.98	0.04	0.07	0.11	0.01	0.28
37980	Philadelphia-Camden-Wilmington, PA-NJ-DE-MD	Philadelphia	1096	0.06	0.19	0.48	0	1	927.25	2153.49	5583.34	6.37	21752.68	0.01	0.06	0.26	0	0.99	0.01	0.02	0.03	0	0.78
38060	Phoenix-Mesa-Scottsdale, AZ	Phoenix	675	0.1	0.36	0.87	0	1	293.8	1238.5	2153.44	0.07	5854.47	0.01	0.02	0.04	0	0.65	0.07	0.14	0.27	0	0.92
38300	Pittsburgh, PA	Pittsburgh	529	0.22	0.43	0.87	0	1	217.74	1442.16	2648.77	10.3	14991.15	0.01	0.02	0.08	0	0.98	0	0	0.01	0	0.04
38900	Portland-Vancouver-Hillsboro, OR-WA	Portland	368	0.19	0.36	0.74	0.02	1	400.75	1216.28	1987.78	0.2	9232.78	0	0.01	0.02	0	0.69	0.02	0.03	0.04	0.01	0.33
39300	Providence-Warwick, RI-MA	Providence	273	0.08	0.17	0.36	0	0.97	935.23	2105.86	3675.23	35.54	12164.41	0.01	0.01	0.03	0	0.45	0.01	0.02	0.05	0	0.41
40140	Riverside-San Bernardino-Ontario, CA	Riverside	608	0.3	0.5	0.79	0.04	0.99	156.28	702.88	1840.87	0.19	6434.01	0.01	0.04	0.09	0	0.51	0.15	0.23	0.34	0.05	0.96
40900	Sacramento--Roseville--Arden-Arcade, CA	Sacramento	337	0.07	0.17	0.79	0.01	1	392.55	1587.56	2361.59	1.08	4946.94	0.01	0.03	0.09	0	0.46	0.07	0.09	0.15	0.02	0.5
41180	St. Louis, MO-IL	Saint Louis	459	0.19	0.41	0.88	0.05	1	207.45	1028.6	2058.49	5.43	5759.58	0.01	0.04	0.26	0	0.99	0.01	0.01	0.01	0	0.28
41700	San Antonio-New Braunfels, TX	San Antonio	337	0.09	0.37	0.86	0	1	216.01	1056.88	1957.63	1.17	3926.26	0.01	0.03	0.07	0	0.83	0.25	0.4	0.72	0.05	0.99
41740	San Diego-Carlsbad, CA	San Diego	468	0.15	0.27	0.49	0.01	1	1273.11	2378.75	3698.62	1.08	10852.04	0.01	0.03	0.07	0	0.73	0.1	0.17	0.3	0.01	0.92
41860	San Francisco-Oakland-Hayward, CA	San Francisco	723	0.02	0.06	0.15	0	0.99	1857.9	3414.58	6173.71	7.1	47138.32	0.02	0.06	0.17	0	0.93	0.07	0.12	0.2	0	0.77
41940	San Jose-Sunnyvale-Santa Clara, CA	San Jose	286	0.08	0.12	0.21	0.01	0.99	1839.87	2904.78	3982.5	0.65	9910.46	0.02	0.03	0.05	0	0.13	0.12	0.19	0.4	0.04	0.82
42660	Seattle-Tacoma-Bellevue, WA	Seattle	538	0.14	0.31	0.57	0	0.94	499.69	1339.28	2064.87	1.23	15680.36	0.01	0.02	0.05	0	0.74	0.02	0.03	0.04	0.01	0.15
45300	Tampa-St. Petersburg-Clearwater, FL	Tampa	548	0.11	0.23	0.53	0.02	0.98	358.99	1175.72	1759.88	8.04	8395.19	0	0.02	0.07	0	0.98	0.02	0.03	0.08	0.01	0.67
47260	Virginia Beach-Norfolk-Newport News, VA-NC	Virginia Beach	305	0.12	0.24	0.45	0.01	0.98	668.47	1494.4	2179.86	5.94	4915.01	0.14	0.24	0.43	0	0.99	0.01	0.02	0.03	0	0.1
47900	Washington-Arlington-Alexandria, DC-VA-MD-WV	Washington	1007	0.2	0.4	0.72	0	1	411.68	1545.72	3173.63	0.51	21205.07	0.07	0.14	0.41	0	1	0.01	0.03	0.07	0	0.51

Table S2. Descriptive statistics for the gentrifiable census tracts within each metropolitan statistical area included in the 2000-2010 period analysis

				% Green 2001					Population density, 2000					% Non-Hispanic Black, 2000					% Hispanic, 2000				
MSA	MSA Name	Core city name	Number of gentrifiable census tracts	Q1	Q2	Q3	Min	Max	Q1	Q2	Q3	Min	Max	Q1	Q2	Q3	Min	Max	Q1	Q2	Q3	Min	Max
12060	Atlanta-Sandy Springs-Roswell, GA	Atlanta	706	0.18	0.34	0.69	0	0.97	177.16	642.09	1406.16	7.3	15808.49	0.08	0.21	0.58	0	0.99	0.02	0.03	0.07	0	0.72
12420	Austin-Round Rock, TX	Austin	256	0.07	0.3	0.74	0	0.95	122.42	926.4	1896.03	3.93	7796.29	0.03	0.06	0.11	0	0.74	0.15	0.23	0.44	0.03	0.87
12580	Baltimore-Columbia-Towson, MD	Baltimore	505	0.01	0.09	0.3	0	0.95	888.55	1960.33	3828.16	13.36	40854.99	0.06	0.2	0.71	0	0.99	0.01	0.01	0.02	0	0.28
14460	Boston-Cambridge-Newton, MA-NH	Boston	746	0	0.07	0.35	0	0.92	686.58	2324.59	6174.03	23.81	46877.51	0.01	0.03	0.08	0	0.92	0.01	0.03	0.11	0	0.88
16740	Charlotte-Concord-Gastonia, NC-SC	Charlotte	396	0.11	0.45	0.8	0	0.96	105.7	315.97	791.11	8.32	4587.63	0.08	0.16	0.33	0.01	1	0.02	0.03	0.06	0	0.44
16980	Chicago-Naperville-Elgin, IL-IN-WI	Chicago	1652	0	0	0.06	0	0.95	1245.94	2916.21	6473.38	7.11	199239.8	0.01	0.05	0.44	0	1	0.03	0.08	0.27	0	0.98
17140	Cincinnati, OH-KY-IN	Cincinnati	372	0.04	0.23	0.57	0	0.98	239.52	1075.11	2123.19	11.84	6856.61	0.01	0.03	0.23	0	0.98	0.01	0.01	0.01	0	0.09
17460	Cleveland-Elyria, OH	Cleveland	474	0	0.01	0.15	0	0.94	899.38	1948.48	3165.93	19.15	9242.3	0.01	0.1	0.62	0	0.99	0.01	0.01	0.03	0	0.57
18140	Columbus, OH	Columbus	325	0	0.07	0.61	0	0.96	179.38	1196.05	2226.48	7.37	9716.08	0.02	0.06	0.22	0	0.95	0.01	0.01	0.02	0	0.43
19100	Dallas-Fort Worth-Arlington, TX	Dallas	980	0	0.07	0.44	0	0.95	365.15	1298.62	2195.06	2.59	22863.32	0.03	0.08	0.19	0	0.97	0.09	0.16	0.31	0.02	0.95
19740	Denver-Aurora-Lakewood, CO	Denver	449	0	0.01	0.15	0	0.99	1086.3	1903.87	2665	0.62	12691.92	0.01	0.02	0.08	0	0.71	0.08	0.13	0.27	0.01	0.84
19820	Detroit-Warren-Dearborn, MI	Detroit	960	0	0	0.08	0	0.94	906.03	1789.14	2683.63	15.53	7424.33	0.01	0.05	0.78	0	0.99	0.01	0.02	0.03	0	0.77
26420	Houston-The Woodlands-Sugar Land, TX	Houston	795	0	0.05	0.38	0	0.97	439.44	1349.07	2350.18	3.95	27361.82	0.04	0.11	0.29	0	0.99	0.15	0.27	0.47	0	0.97
26900	Indianapolis-Carmel-Anderson, IN	Indianapolis	297	0	0.03	0.32	0	0.97	438.56	1009.76	1651.02	9.57	4684.59	0.01	0.05	0.32	0	0.98	0.01	0.02	0.04	0	0.3
28140	Kansas City, MO-KS	Kansas City	387	0	0.11	0.55	0	0.95	251.78	969.84	1580.27	4.74	4361.77	0.02	0.05	0.29	0	0.98	0.02	0.03	0.06	0	0.7
29820	Las Vegas-Henderson-Paradise, NV	Las Vegas	311	0	0	0.05	0	0.99	996.33	2327.83	3357.78	0.27	9238.78	0.04	0.07	0.11	0	0.84	0.09	0.17	0.31	0	0.83
31080	Los Angeles-Long Beach-Anaheim, CA	Los Angeles	2177	0	0	0.01	0	0.97	3100.18	4415.91	7067.85	1.99	48071.79	0.02	0.04	0.1	0	0.94	0.26	0.49	0.74	0.03	0.98
31140	Louisville/Jefferson County, KY-IN	Louisville	222	0.05	0.21	0.56	0	0.98	277.61	1102.53	1772.91	12.17	4395.03	0.02	0.06	0.18	0	0.98	0.01	0.01	0.02	0	0.17
32820	Memphis, TN-MS-AR	Memphis	232	0.01	0.09	0.65	0	0.96	102.27	912.17	1711.35	3.78	4444.57	0.22	0.49	0.88	0.01	1	0.01	0.01	0.03	0	0.18
33100	Miami-Fort Lauderdale-West Palm Beach, FL	Miami	894	0.01	0.03	0.08	0	0.98	1580.53	2460.02	3609.41	2.49	22939.59	0.02	0.08	0.31	0	0.99	0.1	0.22	0.54	0	0.95
33340	Milwaukee-Waukesha-West Allis, WI	Milwaukee	320	0	0	0.09	0	0.92	1129.87	2488.75	4072.7	27.26	11130	0.01	0.05	0.54	0	0.98	0.02	0.04	0.06	0	0.77
33460	Minneapolis-St. Paul-Bloomington, MN-WI	Minneapolis	586	0	0.03	0.31	0	0.95	439.42	1327.67	2262.14	4.6	10597.49	0.01	0.04	0.09	0	0.7	0.01	0.02	0.05	0	0.42
34980	Nashville-Davidson-Murfreesboro-Franklin, TN	Nashville	281	0.15	0.48	0.9	0	0.98	49	378.17	1064.39	4.66	4402.66	0.04	0.1	0.22	0	0.98	0.01	0.02	0.04	0	0.3
35380	New Orleans-Metairie, LA	New Orleans	292	0	0	0.12	0	0.94	922.89	2460.41	3976.84	1.58	29402.05	0.14	0.45	0.87	0	1	0.02	0.03	0.05	0	0.3
35620	New York-Newark-Jersey City, NY-NJ-PA	New York	3460	0	0	0.03	0	0.95	2860.58	9031.85	18701.61	7.31	84839.68	0.02	0.08	0.36	0	0.97	0.07	0.14	0.33	0	0.93
36420	Oklahoma City, OK	Oklahoma City	271	0	0.01	0.43	0	0.96	224.26	1053.7	1606.8	3.41	3458.39	0.03	0.07	0.16	0	0.98	0.03	0.05	0.09	0	0.68
36740	Orlando-Kissimmee-Sanford, FL	Orlando	291	0.11	0.22	0.44	0	0.96	276.03	979.05	1575.21	1.92	6001.61	0.04	0.08	0.17	0	0.97	0.07	0.13	0.21	0.01	0.66
37980	Philadelphia-Camden-Wilmington, PA-NJ-DE-MD	Philadelphia	1096	0	0.08	0.25	0	0.94	876.53	2131.03	5190.51	4.73	24093.62	0.03	0.1	0.35	0	0.98	0.01	0.03	0.05	0	0.88
38060	Phoenix-Mesa-Scottsdale, AZ	Phoenix	684	0	0.05	0.43	0	1	383.64	1516.92	2500.64	0.07	9641.75	0.02	0.03	0.05	0	0.58	0.12	0.24	0.46	0	0.92
38300	Pittsburgh, PA	Pittsburgh	528	0.04	0.19	0.67	0	0.94	176.87	1344.48	2434.23	11.66	11398.18	0.01	0.03	0.12	0	0.99	0	0.01	0.01	0	0.04
38900	Portland-Vancouver-Hillsboro, OR-WA	Portland	368	0	0.04	0.26	0	0.99	503.16	1452.91	2350.74	0.02	9162.1	0.01	0.02	0.03	0	0.53	0.04	0.06	0.09	0	0.59
39300	Providence-Warwick, RI-MA	Providence	273	0.01	0.06	0.21	0	0.87	875.38	2080.79	3837.22	33.12	10220.74	0.01	0.03	0.07	0	0.39	0.02	0.03	0.11	0	0.61
40140	Riverside-San Bernardino-Ontario, CA	Riverside	608	0.02	0.14	0.53	0	0.97	291	1134.85	2290.22	0.23	7761.92	0.02	0.05	0.12	0	0.48	0.22	0.37	0.54	0.04	0.98
40900	Sacramento--Roseville--Arden-Arcade, CA	Sacramento	351	0	0	0.34	0	0.98	462.75	1730.61	2592.48	0.19	6502.81	0.01	0.04	0.12	0	0.39	0.08	0.13	0.22	0.02	0.57
41180	St. Louis, MO-IL	Saint Louis	459	0	0.11	0.61	0	0.96	194.89	985.92	1901.5	3.27	6072.5	0.01	0.06	0.4	0	0.99	0.01	0.01	0.02	0	0.56
41700	San Antonio-New Braunfels, TX	San Antonio	337	0	0.11	0.57	0	0.98	218.86	1202.91	2133.75	1.42	4786.21	0.01	0.04	0.08	0	0.73	0.33	0.54	0.79	0.08	0.98
41740	San Diego-Carlsbad, CA	San Diego	469	0	0.01	0.1	0	0.98	1606.09	2734.22	4072.01	1.16	17254.42	0.02	0.04	0.09	0	0.62	0.13	0.25	0.44	0.03	0.96
41860	San Francisco-Oakland-Hayward, CA	San Francisco	726	0	0	0.02	0	0.93	2135.5	3957.13	7689.89	6.34	64136.14	0.03	0.06	0.17	0	0.8	0.08	0.15	0.26	0.01	0.81
41940	San Jose-Sunnyvale-Santa Clara, CA	San Jose	286	0	0	0	0	0.97	2134.82	3014.97	4434.3	0.63	15239.78	0.02	0.03	0.04	0	0.13	0.14	0.21	0.42	0.04	0.87
42660	Seattle-Tacoma-Bellevue, WA	Seattle	538	0.01	0.08	0.24	0	0.94	665.31	1548.22	2380.13	1.37	18525.08	0.02	0.04	0.09	0	0.54	0.03	0.05	0.07	0.01	0.37
45300	Tampa-St. Petersburg-Clearwater, FL	Tampa	549	0.02	0.11	0.34	0	0.99	378.9	1256.94	1870.66	3.65	8488.24	0.01	0.03	0.11	0	0.97	0.04	0.06	0.12	0	0.71
47260	Virginia Beach-Norfolk-Newport News, VA-NC	Virginia Beach	306	0.02	0.08	0.3	0	0.97	699.75	1498.31	2155.6	5.58	4823.1	0.2	0.3	0.52	0	0.99	0.01	0.03	0.04	0	0.15
47900	Washington-Arlington-Alexandria, DC-VA-MD-WV	Washington	1005	0.04	0.17	0.45	0	0.95	609.46	1787.66	3410.85	7.81	23919.8	0.09	0.18	0.51	0	0.99	0.02	0.05	0.12	0	0.8

Table S3. Spatial autocorrelation of % greenspace, as indicated by the Global Moran's Index		
	Moran's Index	p-value
% greenspace, 1992	0.83	< 0.001
% greenspace, 2001	0.83	< 0.001

Table S4. Meta-analytic estimates of association between % greenspace in 1992 and sociodemographic and housing cost changes, 1990-2000, among gentrifiable census tracts in the 43 largest MSAs in the United States

	% Green in 1992							
	> 50th – 75th percentile				> 75th percentile			
Change variable	Beta	LL	UL	I^2	Beta	LL	UL	I^2
**Unadjusted models**								
% NH Black	0.008	-0.017	0.032	48.90	-0.069	-0.106	-0.031	78.9
% NH White	0.032	0.003	0.062	57.19	0.160	0.118	0.202	79.0
% Hispanic	-0.045	-0.070	-0.020	79.15	-0.133	-0.177	-0.089	94.1
% Bachelor's degree	0.034	-0.003	0.072	59.80	0.207	0.149	0.264	83.3
% Professional jobs	0.071	0.030	0.111	58.66	0.301	0.239	0.363	82.2
% Living in poverty	0.007	-0.037	0.051	54.090	-0.201	-0.269	-0.134	80.7
Median household income	0.029	-0.005	0.063	64.98	0.346	0.285	0.407	88.6
Median home value	-0.033	-0.058	-0.008	70.47	0.081	0.042	0.120	87.8
Median household rent	0.003	-0.021	0.027	51.02	0.131	0.085	0.178	87.5
**Models adjusted for population density in 1990**								
% NH Black	0.019	-0.005	0.043	25.21	0.005	-0.024	0.033	10.3
% NH White	-0.017	-0.042	0.009	18.43	0.027	-0.007	0.062	13.0
% Hispanic	-0.006	-0.026	0.014	43.46	-0.042	-0.073	-0.012	47.8
% Bachelor's degree	-0.029	-0.068	0.010	44.85	0.072	-0.010	0.154	76.3
% Professional jobs	0.017	-0.023	0.058	38.45	0.182	0.105	0.258	65.5
% Living in poverty	0.046	0.002	0.089	33.10	-0.073	-0.155	0.010	62.6
Median household income	-0.009	-0.041	0.023	41.05	0.231	0.151	0.310	81.0
Median home value	-0.043	-0.069	-0.017	58.71	0.055	0.002	0.107	78.4
Median household rent	-0.015	-0.034	0.004	0.00	0.046	0.012	0.081	27.5

Table S5. Meta-analytic estimates of association between % greenspace in 2001 and sociodemographic and housing cost changes, 2000-2010, among gentrifiable census tracts in the 43 largest MSAs in the United States

	% Green 2001			
	> 75th percentile			
Change variable	Beta	LL	UL	I^2
**Unadjusted models**				
% NH Black	0.007	-0.031	0.045	77.1
% NH White	0.042	0.001	0.083	75.2
% Hispanic	-0.090	-0.131	-0.049	72.1
% Bachelor's degree	0.091	0.044	0.139	71.6
% Professional jobs	0.146	0.101	0.190	60.6
% Living in poverty	-0.191	-0.254	-0.127	79.8
Median household income	0.156	0.109	0.202	81.0
Median home value	0.028	-0.006	0.062	85.8
Median household rent	0.074	0.033	0.116	83.9
**Models adjusted for population density in 2000**				
% NH Black	0.030	0.007	0.054	2.8
% NH White	-0.031	-0.079	0.017	56.7
% Hispanic	-0.001	-0.050	0.050	50.0
% Bachelor's degree	-0.095	-0.138	-0.051	20.0
% Professional jobs	-0.029	-0.078	0.021	26.7
% Living in poverty	0.000	-0.045	0.045	12.8
Median household income	0.039	-0.011	0.092	61.9
Median home value	-0.014	-0.052	0.025	68.4
Median household rent	0.027	-0.016	0.072	63.4

Table S6. Meta-analytic estimates of association between % greenspace and sociodemographic and housing cost changes, among gentrifiable census tracts in the 43 largest MSAs in the United States, stratified by % non-Hispanic Black population and adjusted for population density in years 1990 or 2000.[1]

Associations with % green, 1992						
	Low % non-Hispanic Black			High % non-Hispanic Black		
1990-2000 change	Beta	LL	UL	Beta	LL	UL
% NH Black	-0.007	-0.03	0.02	-0.07	-0.14	0.00
% NH White	0.052	0.02	0.08	0.07	0.00	0.15
% Hispanic	-0.035	-0.05	-0.02	-0.02	-0.07	0.02
% Bachelor's degree	0.072	-0.02	0.16	0.09	0.02	0.16
% Professional jobs	0.160	0.08	0.24	0.13	0.07	0.19
% Living in poverty	-0.089	-0.14	-0.03	-0.12	-0.23	-0.02
Median household income	0.235	0.15	0.32	0.18	0.11	0.25
Median home value	0.105	0.06	0.15	0.05	0.00	0.09
Median household rent	0.071	0.02	0.12	0.05	0.01	0.10

Associations with % green, 2001						
	Low % non-Hispanic Black			High % non-Hispanic Black		
2000-2010 change	Beta	LL	UL	Beta	LL	UL
% NH Black	0.019	-0.02	0.06	0.077	0.02	0.14
% NH White	0.001	-0.06	0.06	-0.102	-0.16	-0.04
% Hispanic	-0.006	-0.06	0.05	0.026	-0.04	0.09
% Bachelor's degree	-0.071	-0.13	-0.02	-0.157	-0.22	-0.09
% Professional jobs	0.009	-0.04	0.05	-0.117	-0.17	-0.06
% Living in poverty	-0.009	-0.06	0.04	-0.003	-0.08	0.07
Median household income	0.050	0.00	0.10	0.003	-0.07	0.08
Median home value	-0.015	-0.05	0.02	-0.028	-0.08	0.02
Median household rent	0.057	-0.01	0.12	-0.029	-0.08	0.02

[1]Beta coefficients represent the comparison of the census tracts with % greenspace > 75th percentile of the MSA-distribution vs. tracts with % greenspace <= the 75th percentile

Table S7. Meta-analytic estimates of association between % greenspace and sociodemographic and housing cost changes, among gentrifiable census tracts in the 43 largest MSAs in the United States, stratified by % Hispanic population and adjusted for population density in years 1990 or 2000.[1]

	% Green 1992					
	Low % Hispanic			High % Hispanic		
Change, 1990-2000	Beta	LL	UL	Beta	LL	UL
% NH Black	-0.010	-0.04	0.02	-0.01	-0.05	0.03
% NH White	-0.006	-0.04	0.02	0.10	0.05	0.15
% Hispanic	-0.012	-0.03	0.01	-0.08	-0.12	-0.03
% Bachelor's degree	0.093	0.01	0.18	0.10	0.00	0.19
% Professional jobs	0.153	0.08	0.23	0.16	0.09	0.24
% Living in poverty	-0.058	-0.12	0.00	-0.12	-0.20	-0.04
Median household income	0.235	0.16	0.31	0.20	0.11	0.29
Median home value	0.115	0.07	0.16	0.05	0.00	0.09
Median household rent	0.073	0.03	0.11	0.07	0.01	0.14
	% Green 2001					
	Low % Hispanic			High % Hispanic		
Change, 2000-2010	Beta	LL	UL	Beta	LL	UL
% NH Black	0.038	-0.01	0.08	0.05	0.00	0.09
% NH White	-0.037	-0.09	0.02	-0.05	-0.11	0.01
% Hispanic	0.037	-0.02	0.09	-0.03	-0.10	0.05
% Bachelor's degree	-0.093	-0.15	-0.04	-0.10	-0.16	-0.05
% Professional jobs	-0.025	-0.09	0.04	-0.04	-0.11	0.03
% Living in poverty	0.018	-0.03	0.07	-0.03	-0.10	0.04
Median household income	0.042	-0.01	0.10	0.04	-0.03	0.11
Median home value	-0.002	-0.04	0.04	-0.04	-0.09	0.00
Median household rent	0.020	-0.04	0.08	0.03	-0.02	0.08

[1]Beta coefficients represent the comparison of the census tracts with % greenspace > 75th percentile of the MSA-distribution vs. tracts with % greenspace <= the 75th percentile

Table S8. Sensitivity analysis results showing meta-analytic estimates of association between % greenspace in 1992 and sociodemographic and housing cost changes, 1990-2000.

	Original analysis results				Use of rook rather than Queens matrix				Inclusion of all census tracts, regardless of eligibility to gentrify				Restricted to census tracts that had a median household income >= 50th percentile of their MSA			
	> 50% - 75%		> 75 %		> 50% - 75%		> 75 %		> 50% - 75%		> 75 %		> 50% - 75%		> 75 %	
Change variable	Beta	SE	Beta	SE	Beta	SE	Beta	SE	Beta	SE	Beta	SE	Beta	SE	Beta	SE
% NH Black	0.01	0.01	-0.07	0.02	0.01	0.02	-0.06	0.03	0.00	0.01	-0.06	0.02	0.01	0.02	-0.06	0.03
% NH White	0.03	0.01	0.16	0.02	0.02	0.02	0.16	0.03	0.05	0.01	0.13	0.01	0.02	0.02	0.16	0.03
% Hispanic	-0.04	0.01	-0.13	0.02	-0.02	0.01	-0.13	0.02	-0.06	0.01	-0.11	0.02	-0.02	0.01	-0.13	0.02
% Bachelor's degree	0.03	0.02	0.21	0.03	0.01	0.02	0.11	0.03	0.09	0.02	0.28	0.02	0.01	0.02	0.11	0.03
% Professional jobs	0.07	0.02	0.30	0.03	0.03	0.02	0.21	0.03	0.12	0.02	0.34	0.03	0.03	0.02	0.21	0.03
% Living in poverty	0.01	0.02	-0.20	0.03	0.01	0.02	-0.20	0.03	-0.01	0.02	63.60	-0.19	0.01	0.03	-0.19	0.04
Median household income	0.03	0.02	0.35	0.03	0.00	0.02	0.26	0.03	0.07	0.02	0.41	0.03	0.00	0.02	0.26	0.03
Median home value	-0.03	0.01	0.08	0.02	-0.04	0.01	0.02	0.02	-0.01	0.01	0.11	0.02	-0.04	0.01	0.02	0.02
Median household rent	0.00	0.01	0.13	0.02	-0.01	0.01	0.08	0.02	0.02	0.02	0.17	0.03	-0.01	0.01	0.08	0.02

Table S9. Sensitivity analysis results showing meta-analytic estimates of association between % greenspace in 2001 and sociodemographic and housing cost changes, 2000-2010.

	Original analysis results		Use of rook rather than Queens matrix		Inclusion of all census tracts, regardless of eligibility to gentrify		Restricted to census tracts that had a median household income >= 50th percentile of their MSA	
	> 75 %		> 75 %		> 75 %		> 75 %	
Change variable	Beta	SE	Beta	SE	Beta	SE	Beta	SE
% NH Black	0.007	0.020	0.010	0.020	0.006	0.016	0.029	0.019
% NH White	0.042	0.021	0.040	0.021	0.027	0.018	0.008	0.023
% Hispanic	-0.090	0.021	-0.092	0.022	-0.080	0.014	-0.038	0.028
% Bachelor's degree	0.091	0.024	0.092	0.025	0.126	0.022	0.026	0.023
% Professional jobs	0.146	0.023	0.149	0.023	0.151	0.022	0.090	0.025
% Living in poverty	-0.191	0.032	-0.195	0.033	-0.166	0.024	-0.115	0.038
Median household income	0.156	0.024	0.157	0.024	0.129	0.025	0.089	0.022
Median home value	0.028	0.017	0.027	0.018	0.044	0.017	-0.019	0.018
Median household rent	0.074	0.021	0.077	0.022	0.096	0.026	0.031	0.018
